# Jasmonic Acid (JA) Acts as a Signal Molecule in LaCl_3_-Induced Baicalin Synthesis in *Scutellaria baicalensis* Seedlings

**DOI:** 10.1007/s12011-012-9379-8

**Published:** 2012-04-03

**Authors:** Jie Zhou, Lei Fang, Xuan Li, Lanping Guo, Luqi Huang

**Affiliations:** 1 Shandong Analysis and Test Center, Shandong Academy of Sciences, Jinan, 250014 China; 2 Institute of Chinese Materia Medica, China Academy of Chinese Medical Sciences, Beijing, 100700 China

**Keywords:** LaCl_3_, Jasmonic acid, *Scutellaria baicalensis*, Baicalin

## Abstract

Rare earth elements (REEs) have been widely used to increase accumulation of biomass and secondary metabolites in medicinal plants in China. However, very few studies have investigated how REEs mediate secondary metabolism synthesis in medicinal plants. Lanthanum (La), an important REE, is known to improve the accumulation of secondary metabolites in medicinal plants and is widely distributed in China. However, few studies have evaluated the signal transduction leading to La-induced secondary metabolism in medicinal plants. In this study, LaCl_3_ treatment-induced multiple responses in *Scutellaria baicalensis* seedlings, including the rapid generation of jasmonic acid (JA), sequentially followed by the enhancement of baicalin production. Direct application of JA also promoted the synthesis of baicalin in the absence of LaCl_3_. LaCl_3_-induced baicalin synthesis was blocked by two different JA synthesis inhibitors. Our results showed that JA acts as a signal component within the signaling system leading to La-induced baicalin synthesis in *S. baicalensis* seedlings.

## Introduction

Traditional Chinese medicine (TCM) plays an important role in maintaining people’s health [1]. During the last two decades, the use of herbal medicines has expanded globally and gained considerable attention because of their low toxicity and good therapeutic performance [2]. *Scutellaria baicalensis* (referred to in TCM as “huang qin”) is one of the 50 fundamental herbs used in TCM. Baicalin is the most abundant active component in huang qin and is an important secondary metabolite in *S. baicalensis* induced by exogenous stress. It has anti-inflammatory, anti-HIV, anti-tumor and anti-severe acute respiratory syndrome coronavirus effects [3]. The ever-increasing demand for huang qin has stimulated the improvement of cultivated practices of *S. baicalensis*.

Rare earth elements (REEs), which comprise elements in the lanthanide series from lanthanum (La) to lutetium (Lu) and commonly also include scandium (Sc) and yttrium (Y), have been widely distributed in China and been considered as “strategic element” [4]. REEs have been reported to increase biomass and secondary metabolite synthesis in medicinal plants. Many studies on the physiological effects of REE application have shown that they could improve photosynthesis significantly by promoting the absorption, transfer, and transformation of light energy and photochemical activity in chloroplasts, promote nitrogen metabolism [5–11], as well as replace calcium in functions such as regulating stomatal movement [12]. However, very few studies have investigated how REEs mediate secondary metabolism synthesis in medicinal plants.

Jasmonic acid (JA) is an important signaling molecule involved in elicitor-induced secondary metabolite synthesis (such as terpenoids, flavonoids, alkaloids) [13]. REEs, as exogenous elicitors, may stimulate the defense system of plant cells and promote the accumulation of secondary metabolites [14]. However, we are aware that no published studies that confirmed that JA mediates REE-induced secondary metabolite biosynthesis in medicinal plants.

La, a representative REE, is one of the most abundant REEs in China. In this study, we investigated (1) the effects of La on endogenous JA levels and baicalin biosynthesis in *S. baicalensis* seedlings and (2) the effects of exogenous JA and its synthesis inhibitors on baicalin biosynthesis to further understand the mechanisms by which REEs improve secondary metabolite production in *S. baicalensis* seedlings.

## Materials and Methods

### Plant Culture and Treatment

Seeds of *S. baicalensis* were surface decontaminated with 0.1 % mercuric chloride for 2 min, rinsed with distilled water, then sown in a twice-autoclaved mixture of garden soil and river sand (1:1, *v*/*v*). After emergence of the sixth leaf, uniform-sized seedlings were washed with distilled water and transferred into pots filled with quartz sand. Half-strength Hoagland’s solution was used as a liquid growth medium. Seedlings were incubated in a greenhouse at 25/22°C day/night under a 14-h photoperiod (220 ± 5 μmol/m^2^·s) at a relative humidity of 65 %. After 2 weeks of growth (a time sufficient to minimize the stress factors from the change in nutrient medium), plants were treated with a solution of either (1) LaCl_3_ (100 mg/L), (2) jasmonic acid methyl ester (JAMe; 10^−5^ mol/L), (3) LaCl_3_ and its synthesis inhibitor salicylhydroxamic acid (SHAM; 100 μmol/L), (4) LaCl_3_ and its synthesis inhibitor *n*-propyl gallate (PrGall) (100 μmol/L), and (5) JAMe and SHAM. Six plants were used in each treatment. Solutions were sprayed evenly on leaves until excess drops began to fall. SHAM was dissolved in minimal amounts of DMSO (0.1 mmol/L SHAM in 20 μL DMSO) and JAMe and PrGall in ethanol. Equal amounts of DMSO and ethanol were sprayed on control plants. SHAM (100 μmol/L), and PrGall (100 μmol/L) were added 45 min before the treatment with LaCl_3_ or JAMe. All solutions were determined in the pre-experiments. The leaves and roots were harvested, frozen in liquid nitrogen, and then kept at −80°C for all subsequent analyses.

### Determination of JA

Extraction, purification, and determination of endogenous levels of JA by an indirect enzyme-linked immunosorbent assay technique were performed as described by Yang et al. [15].

### Determination of Baicalin

Quantitative analysis of baicalin in *S. baicalensis* seedlings was performed according to the reported procedure [16]. Briefly, the air-dried roots of individual *S. baicalensis* plants (500 mg) were ground into power (20 mesh), soaked in 75 % ethanol (100 mL), and sonicated (300 W, 25 kHz) for 30 min. The extract was filtered through a 0.45 μm membrane filter, and 10 μL was injected for each HPLC analysis. HPLC analysis was performed on a Kinetex C_18_ (4.6 × 100 mm, 2.6 μm) column. The detection wavelength was set at 275 nm and the column component was maintained at 40°C. The mobile phase consisted of A (1 % tetrahydrofuran), B (acetonitrile), and C (5 % methanoic acid), using a gradient of A (68.5–63.0 %), B (14.5–17.0 %), and C (17.0–20 %) from 0 to 13 min; A (63.0–48.0 %), B (17.0–32.0 %), and C (20–20 %) from 13 to 28 min; A (48.0–38.0 %), B (32.0–42.0 %), and C (20–20 %) from 28 to 40.5 min; A (38.0–0 %), B (42.0–80 %), and C (20–20 %) from 40.5 to 43 min; and A (0–68.5 %), B (80.0–14.5 %), and C (20–17 %) from 43 to 45 min. The flow rate was 1.3 ml/min, and the contents of the bioactive components were calculated from corresponding linear relationships for peak area concentration.

### Statistical Analyses

The estimated values were the means of samples ± standard deviation of the mean. Significant differences were determined by one-way ANOVA test using SPSS v. 13. Differences were considered significant at *p* < 0.05.

## Results

### LaCl_3_-Induced JA Generation in *S. baicalensis* Seedlings

**Fig. 1:**
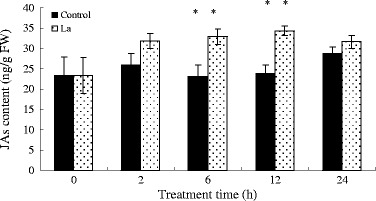
JA content of *S. baicalensis* seedlings over 24 h after treatment with LaCl_3_. *Vertical bars* represent the mean ± SD (*n* = 6). Means with *asterisks* indicate significant differences at *p* < 0.05

As shown in Fig. 1, *S. baicalensis* seedlings responded to LaCl_3_ treatment by rapidly generating JA. JA levels in *S. baicalensis* seedlings reached 122.63 % of the control (*p* > 0.05) within 2 h of addition of LaCl_3_. Treated plants had 42.12 % more JA than control plants by 6 h, a significant difference (*p* < 0.05), and 43.87 % more (*p* < 0.05) by 12 h after LaCl_3_ application. By 24 h after treatment, JA levels had degraded and were not significantly different from the control. Overall, LaCl_3_ resulted in a JA burst in the 24 h after the treatment.

### LaCl_3_-Induced Baicalin Synthesis in *S. baicalensis* Seedlings

**Fig. 2:**
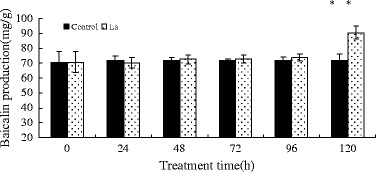
Effects of LaCl_3_ treatment on baicalin production in *S. baicalensis* seedlings. *Vertical bars* represent the mean ± SD (*n* = 6). Means with *asterisks* indicate significant differences at *p* < 0.05

Figure 2 shows that LaCl_3_ treatment resulted in an increase in baicalin content in *S. baicalensis* seedlings. The LaCl_3_-induced increase in baicalin production occurred mainly after the JA peak, peaking at 1.25-fold of control plant levels after 5 days of treatment (*p* < 0.05).

### Exogenous JA-Enhanced Baicalin Synthesis in *S. baicalensis* Seedlings in Absence of LaCl_3_

**Fig. 3:**
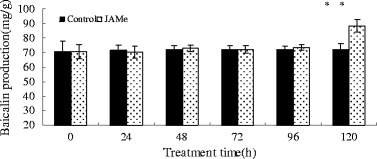
Effects of JAMe treatment on baicalin production in *S. baicalensis* seedlings. *Vertical bars* represent the mean ± SD (*n* = 6). Means with *asterisks* indicate significant differences at *p* < 0.05

JAMe was used in this work to investigate the effects of exogenous JA on baicalin production in the absence of LaCl_3_ (Fig. 3). JAMe stimulated baicalin production, exceeding as much as 90 % of the LaCl_3_ response after 5 days of treatment.

### La-Induced Baicalin Production was Blocked by JA Synthesis Inhibitors

**Fig. 4:**
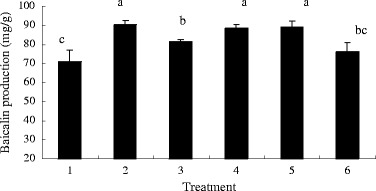
Effects of LaCl_3_, JAMe, and JA synthesis inhibitors on baicalin production in *S. baicalensis* seedlings. *1* Control, *2* LaCl_3_, *3* LaCl_3_ + SHAM, *4* LaCl_3_ + PrGall, *5* JAMe, *6* JAMe + SHAM *S. baicalensis* seedlings were treated with LaCl_3_ (100 mg/L) and JAMe (10^−5^ mol/L). SHAM (100 μmol/L) and PrGall (100 μmol/L) were added 45 min before the treatment with LaCl_3_ or JAMe. The control received the same volumes of vehicle solvents. Baicalin production was determined after 5 days. *Vertical bars* represent the means ± SD (*n* = 6). Means with *asterisks* indicate significant differences at *p* < 0.05

The effects of LaCl_3_, JAMe, and JA synthesis inhibitors on baicalin production in *S. baicalensis* seedlings were shown in Fig. 4. The La-induced baicalin synthesis was significantly (*p* < 0.05) blocked by SHAM, an inhibitor of JA synthesis. Although JA synthesis was inhibited by SHAM in the presence of LaCl_3_, baicalin production was significantly higher than the control (*p* < 0.05). The JA synthesis inhibitor PrGall also suppressed the La-induced increase of baicalin (*p* > 0.05).

## Discussion

Our data indicated a connection between LaCl_3_, JA, and baicalin production in *S. baicalensis* seedlings. LaCl_3_ treatment resulted in a JA burst and an increase in baicalin production. La-induced JA generation occurred earlier than the activation of baicalin synthesis, which indicated that JA might be generated as a signal prior to La-induced baicalin synthesis. Direct application of JA also induced baicalin synthesis, suggesting exogenous JA alone can induce baicalin synthesis in *S. baicalensis* seedlings. This further indicated that JA is an intermediate signal to induce baicalin synthesis. The La-induced baicalin synthesis was blocked by JA synthesis inhibitors, which strongly suggested that JA was involved in the LaCl_3_ signal transduction that induced baicalin synthesis, in other words, baicalin synthesis was induced at least partially via a JA signal transduction pathway. Although the JA synthesis was suppressed by SHAM in the presence of LaCl_3_, baicalin production was significantly higher than in the control, implying that JA was not the only signal molecule for inducing baicalin synthesis. LaCl_3_ may have induced baicalin synthesis through other signal transduction pathways when JA signal transduction was impaired in *S. baicalensis* seedlings. The JA synthesis inhibitor PrGall also suppressed the La-induced increase of baicalin, further supporting the presence of another signal molecule via which La-induced baicalin accumulation.

REEs are considered important exogenous elicitors and used to stimulate secondary metabolite systems in medicinal plants [14]. Thus far, however, very few studies have investigated the signal transduction pathways of REE-induced secondary metabolite synthesis. JA biosynthesis is a well-characterized reaction of plants subjected to exogenous elicitors. JA signaling was reported to be involved in elicitor-induced flavonol glycoside accumulation [17]. However, the biosynthetic pathways for secondary metabolite production are regulated by multiple signal elements, such as JA, salicylic acid, reactive oxygen species, and nitric oxide, and their signal pathways. Our results demonstrated that although JA acts as a component of a signaling system leading to La-induced baicalin synthesis, it was not the only signal molecule for inducing baicalin synthesis.

## Conclusions

The data obtained from the present work demonstrated that JA acted as a component of the signaling system leading to La-induced baicalin synthesis in *S. baicalensis* seedlings. Further understanding of the signal transduction pathway of REEs-induced secondary metabolite synthesis will be enhanced by ongoing efforts to elucidate these systems.
